# Peripheral/central neural microstructural alterations in persistent idiopathic dentoalveolar pain: a case-comparative study

**DOI:** 10.3389/fneur.2026.1905273

**Published:** 2026-07-23

**Authors:** Motoko Watanabe, Keigo Shimoji, Junichiro Sakamoto, Chizuko Maeda, Kiyokazu Iwawaki, Gayatri Nayanar, Qinyi Chen, Chihiro Takao, Risa Tominaga, Yasuyuki Kimura, Atsushi Ito, Trang Hu Thi Tu, Masahiko Miura, Akihiko Wada, Akira Toyofuku

**Affiliations:** 1 Department of Psychosomatic Dentistry, Graduate School of Medical and Dental Sciences, Institute of Science Tokyo, Tokyo, Japan; 2 Department of Radiology, Juntendo University Graduate School of Medicine, Tokyo, Japan; 3 Department of Dental Radiology and Radiation Oncology, Graduate School of Medical and Dental Sciences, Institute of Science Tokyo, Tokyo, Japan; 4 Faculty of Dentistry, Department of Basic Dental Science, University of Medicine and Pharmacy at Ho Chi Minh City, Ho Chi Minh City, Vietnam

**Keywords:** atypical odontalgia, central sensitization, diffusion tensor image, magnetic resonance image, neurovascular compression, pain catastrophizing, persistent idiopathic dentoalveolar pain, somatic symptom burden

## Abstract

Persistent idiopathic dentoalveolar pain (PIDAP) has been considered to involve the predominant peripheral or central nervous system, but details of mechanisms remain unclear. This study investigated differences in clinical characteristics and microstructural integrity at the peripheral trigeminal nerve and central white matter between patients with PIDAP, depending on the presence or absence of neurovascular compression (NVC) at the root entry zone (REZ). This retrospective case-comparative study enrolled patients with PIDAP who underwent diffusion tensor imaging to evaluate neural integrity by fractional anisotropy (FA). FA of the trigeminal nerve at the REZ was assessed as a peripheral interaction, and FA in the white matter was assessed as a central interaction. Among 27 patients with NVC and 39 without NVC, pain intensity was significantly higher in patients without NVC (44.3 ± 27.5; 58.6 ± 25.4; *p* = 0.024). While patients with NVC showed significant FA reduction in the trigeminal nerve, patients without NVC showed a tendency toward FA reduction in the white matter. Right- and left-asymmetric FA patterns were detected in pain-related brain regions regardless of the presence of NVC. Somatic symptoms scale-8 (SSS-8) scores were negatively correlated with FA in the white matter in patients with NVC, whereas in patients without NVC, FA in pain-related brain regions was positively correlated with clinical characteristics: pain intensity, pain catastrophizing scales, and SSS-8 scores. These results suggest that PIDAP may have subgroups with peripheral- or central-predominant mechanisms, depending on the presence of NVC. While PIDAP with NVC may relate to FA reduction in the peripheral trigeminal nerve at REZ and the central microstructural alterations related to somatic symptom burden, PIDAP without NVC may be associated with FA alterations in pain-related brain regions, relating to pain severity, without peripheral neural alterations. Although further investigations are required, asymmetric FA patterns in certain pain-related brain regions could be specific features of PIDAP, regardless of the presence of NVC.

## 1 Introduction

Persistent idiopathic dentoalveolar pain (PIDAP), a persistent dentoalveolar pain without corresponding abnormalities (1), was previously known as atypical odontalgia, which has also been categorized as a subtype of persistent idiopathic facial pain (PIFP) (2). The etiology of PIDAP has been considered in relation to both the peripheral and central nervous systems. From a peripheral perspective, nociception may be involved. Dental procedures, such as root canal treatments and tooth extractions, can occasionally be onset triggers of PIDAP, with a reported prevalence of 56.7% (3). Specifically, neurovascular compression (NVC) at the root entry zone (REZ) has been investigated as a potential contributor to neuropathic pain in the orofacial region (4). The trigeminal REZ, located within 3–5 mm of the pons, is the transition zone from peripheral schwannoma cell myelination to central oligodendroglia myelination and is vulnerable to blood vessels (5, 6). NVC of the trigeminal nerve at REZ causes local neural alteration related to orofacial pain and plays a crucial role in relaying sensory signals between the central and peripheral nervous systems (4). In addition to these previous findings, because central nervous factors have also been considered for PIDAP, we have focused on NVC to investigate both peripheral and central involvement in PIDAP.

Our previous research revealed that 40.5% of patients with PIDAP presented NVC, including neurovascular contact, at REZ, and the patients without NVC exhibited significantly higher pain intensity compared to those with NVC (7). Similarly, in a study of patients with oral cenesthopathy presenting with unpleasant oral sensations, NVC was detected in 45.6% of patients, and patients without NVC had higher severity of oral sensations (8). NVC, including neurovascular contact, helps differentiate clinical characteristics of these complex oral sensations. Particularly, PIDAP can be categorized into two distinct subgroups, with different clinical features, including pain characteristics and severity, by the presence of NVC at REZ. Moreover, Maarbjerg et al. reported that while some patients with PIFP presented with NVC, it was less severe than in patients with trigeminal neuralgia (9), indicating different underlying mechanisms. In addition to the severity of NVC, central sensitization and somatic symptom burden are useful in differentiating PIDAP and TN with persistent pain (10). This indicates a complex interaction between peripheral and central mechanisms underlying NVC in patients with indeterminate dental pain. Studies investigating the relationship between NVC and neural alterations have demonstrated decreased neural integrity in both the trigeminal nerve at REZ and various brain regions, including the corpus callosum, cingulum bundle, corona radiata, and superior longitudinal fasciculus in patients with trigeminal neuralgia (4). While decreased neural integrity with NVC in the trigeminal nerve at REZ may relate to the integrity of chronic orofacial pain, it is also associated with brain white matter alteration (4). However, the nature of microstructural alterations in the peripheral trigeminal nerve and the central nervous system in PIDAP remains to be fully elucidated (11).

Based on these observations, we hypothesized a potential association between the presence of NVC and differences in clinical characteristics and microstructural integrity of the peripheral and central nervous systems. Specifically, we anticipated that patients with PIDAP with NVC would present with lower pain intensity and demonstrate reduced fractional anisotropy (FA) in the peripheral trigeminal nerve, while those without NVC would exhibit more severe pain and microstructural alterations in central pain-processing regions. To investigate this hypothesis, we used diffusion tensor imaging (DTI), a magnetic resonance imaging (MRI) technique that quantifies and images water-molecule diffusion direction and capacity, allowing evaluation of white matter integrity through FA measurements. This study aimed to clarify differences in clinical characteristics, including pain intensity and psychological factors, as well as radiological findings, between patients with PIDAP with and without NVC.

## 2 Methods

### 2.1 Participants

Inclusion criteria were the outpatients from January 2017 to January 2023, (1) who were diagnosed with PIDAP, which presents persistent intraoral dentoalveolar pain, recurring daily for more than 2 h per day for more than 3 months, without any preceding causative event, according to International Classification of Orofacial Pain, first edition (ICOP) (1), (2) who showed PIDAP symptoms unilaterally: only on the right or left side, and (3) who underwent MRI examination taking DTI within a month from the first clinical examination. The outpatients before the publication of ICOP, first edition, were initially diagnosed with atypical odontalgia as a subtype of PIFP according to the International Classification of Headache Disorders, third edition (2), or its beta-version (12), and retrospectively confirmed their diagnosis of PIDAP by ICOP, 1st edition. Exclusion criteria were (1) those who were younger than 18 years old, (2) who had a better accounted for by another ICOP, first edition, or the International Classification of Headache Disorders, third edition diagnosis, including any organic brain disorders, (3) who presented PIDAP symptoms bilaterally, and (4) who had a contraindication to taking an MRI.

All clinical and demographic data at the first clinical examination, including sex, age, pain duration, pain intensity, dental procedure as an onset trigger, pain distribution, including affected laterality, psychiatric comorbidities, medication status, and the results of questionnaires described in the following section, were retrospectively acquired from clinical medical charts.

The participants were binary categorized depending on the presence of NVC into two groups: patients with PIDAP with NVC and without NVC. This study followed STROBE guidelines and was approved by the Ethics Committee of the Institute of Science, Tokyo, Faculty of Dentistry (approval No. D2021-091).

### 2.2 Clinical assessments

For clinical assessment, the short-form of the McGill Pain Questionnaire (SF-MPQ) (13), including the visual analog scale (VAS, 0: no pain to 100: approximately severe pain), was used to measure pain characteristics and intensity. Zung’s self-rating depression scale (SDS) (14) was used to assess their depressive state, and the pain catastrophizing scale (PCS) was used to assess their pain catastrophizing (15, 16). Moreover, the Somatic Symptom Scale-8 (SSS-8) was used to assess the somatic symptom burden (17, 18). The presence of psychiatric comorbidities was assessed based on diagnoses made by attending medical doctors, including psychiatrists, through medical chart review. When psychiatric comorbidities were reported by patients, consultations with their attending doctors were conducted to confirm their diagnosis and the conditions of psychiatric disorders.

### 2.3 MRI acquisition

All MR images were obtained with a 3-Tesla MRI scanner (Magnetom Spectra, Siemens Healthcare, Erlangen, Germany) and a 16-channel head coil. For the examination at the REZ level of the trigeminal nerve, MR sequences were acquired using the same parameters as those in our previous study (7, 8, 10). MR cisternography was obtained through 3D constructive interference in steady-state (3D-CISS) using the following parameters: repetition time (TR)/ echo time (TE), 7.4/3.7 ms; flip angle, 50°; FOV, 160 × 160 mm; matrix, 320 × 320; section thickness, 0.5 mm; which was reconstructed to a voxel size of 0.5 × 0.5 × 0.5 mm and a slab thickness of 44 mm. MR angiography (MRA) was obtained using 3D time-of-flight (3D-TOF) MRA with the following parameters: repetition time (TR)/echo time (TE), 24/3.9 ms; flip angle, 18°; field of view (FOV), 160 mm × 160 mm; matrix, 320 × 192; section thickness, 0.5 mm; and slab number, 3. This was reconstructed to a voxel size of 0.5 mm × 0.5 mm × 0.5 mm and a slab thickness of 44 mm. DTI was acquired using the parameters: TR/TE, 5600/94; flip angle, 90°; FOV, 256 × 256 mm; matrix, 128 × 128; slice thickness, 4 mm; pixel spacing, 2 × 2 mm.

### 2.4 Assessment of NVC

**Figure 1:**
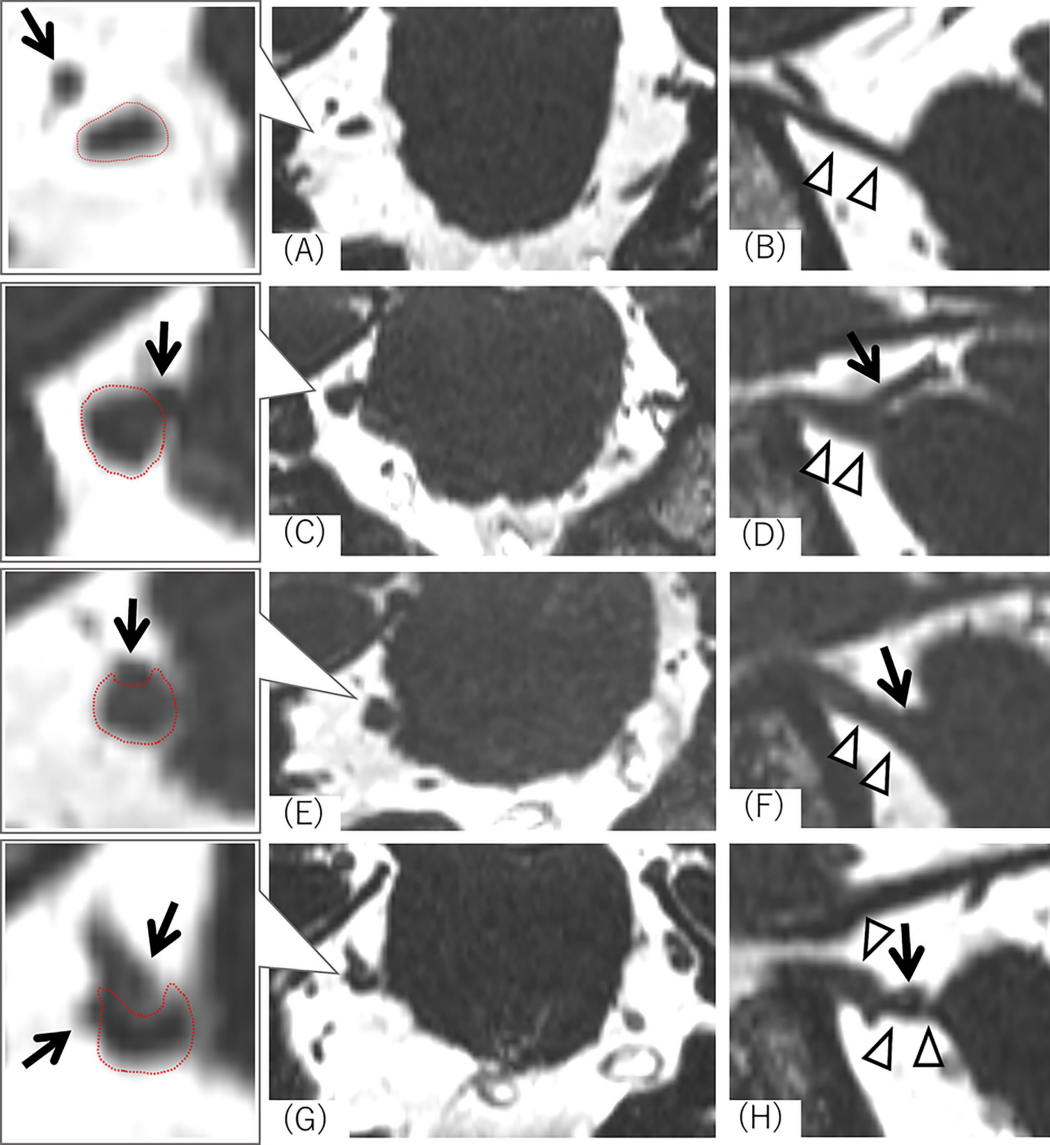
Images of detailed NVC conditions. **(A,B)**: without NVC, **(C,D)**: contact, **(E,F)**: compression, and **(G,H)**: displacement. **(A,C,E,G)**: axial images, **(B,D,F,H)**: sagittal images of the trigeminal nerve. The detailed NVC conditions were assessed by considering the shape (outlined in red dotted line) and course (white arrowheads) of the trigeminal nerve in relation to the three-dimensional spatial arrangement of the blood vessels (black arrows).

All 3D-CISS images were presented in triplanar views (transverse, coronal, and sagittal views) on the visualization system. NVC presence was defined as contact between a blood vessel and the trigeminal nerve at REZ, where 5 mm is at the root of the trigeminal nerve from the pons, with no visible cerebrospinal fluid between them in the tri-planar 3D-CISS images. The blood vessel responsible for NVC was assessed using 3D-TOF MRA. Disagreements or uncertainties regarding whether there was contact were indicated as ‘without NVC’ according to the clinical imaging diagnosis. As detailed in the NVC conditions, simple contact, compression, and displacement were also assessed by the shape and course of the trigeminal nerve (Figure 1). Compression due to lesions (i.e., auditory nerve tumors or trigeminal schwannomas of the trigeminal nerve) was carefully reviewed and excluded. Two experienced oral radiologists, blinded to the symptom side, evaluated the presence of NVC and detailed its condition.

### 2.5 DTI data processing and analysis

**Figure 2:**
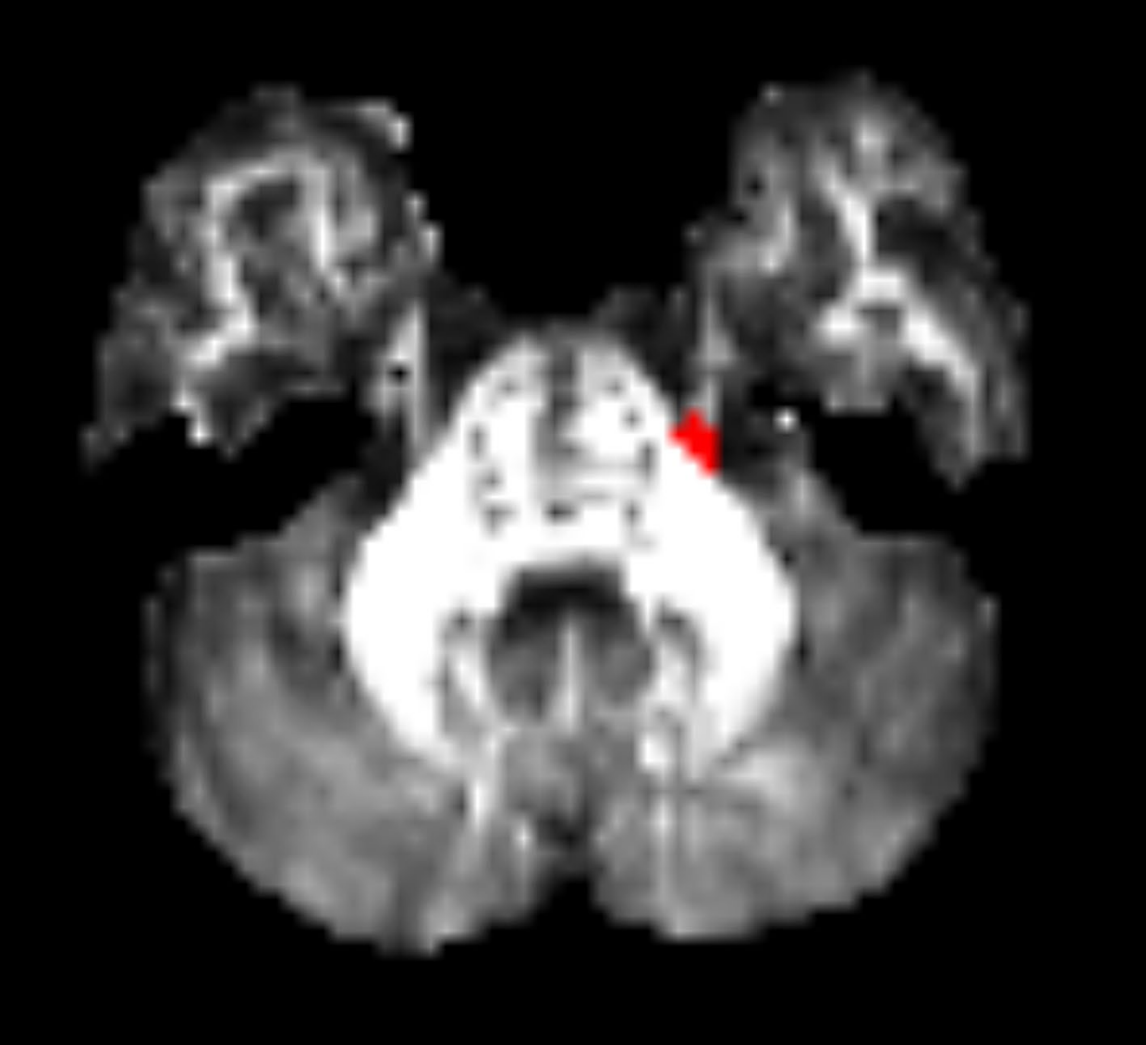
Region of interest (ROI) of the trigeminal nerve at the root entry zone (REZ). The ROI was manually drawn using MRcron to measure fractional anisotropy in the trigeminal nerve at REZ, with 6–8 voxels, including the base of the trigeminal nerve at the pons.

All MRI data were transferred using MRcron and preprocessed using MRtrix. Following denoising, Gibbs artifacts were removed. To correct distortion, topup and eddy in FSL were performed, and bias-field correction was also performed; then, masks were generated. After converting the format, DTI maps were generated. MRcron was also used to draw the region of interest (ROI) manually on the trigeminal nerve at REZ. ROIs were designed with 6 to 8 voxels, minimizing the influence of FA in cerebrospinal fluid, including the base of the trigeminal nerve in the pons (Figure 2). The FA average of voxels in the ROI was then calculated.

For whole-brain analysis, voxel-wise statistical analysis of the FA data was performed using Tract-Based Spatial Statistics (TBSS), implemented in FSL (19, 20). All FA images were non-linearly registered to the standard-space FA template (FMRIB58_FA_1 mm) (21). The aligned FA image for each participant was then projected onto the mean FA skeleton generated with an FA threshold of 0.2. Between-group differences in skeletonized FA were assessed using permutation-based non-parametric inference with the FSL randomize tool. An unpaired t-test was performed with 5,000 permutations, threshold-free cluster enhancement (TFCE), and family-wise error (FWE) correction for multiple comparisons across voxels. Statistical significance was defined as a TFCE-based FWE-corrected *p*-value of < 0.05. Statistical maps showing FWE-corrected significant results were visualized using FSLeyes.

Following TBSS, ROI-based analysis and analysis for each tract of FA were performed using a standardized atlas: Johns Hopkins University White Matter Labeled/Tractography Atlases (22). Bonferroni correction was conducted following between-group analysis in right/left or affected/non-affected sides FA asymmetry using a paired t-test. The partial correlation analysis between FA in each tract and brain region and the scores of questionnaires: SDS, PCS, SSS-8, and VAS (controlled variables: sex, age, pain duration, and the presence of psychiatric comorbidities) was conducted, followed by Bonferroni correction.

### 2.6 Statistical analysis

All statistical analyses were performed using SPSS Statistics version 29 (IBM Corporation, Armonk, NY, USA). A chi-squared test was performed for the analysis of between-group differences in sex, the presence of psychiatric comorbidities, and dental procedure as an onset trigger, and an unpaired t-test was performed for age, pain duration, and the results of questionnaires, with the statistical significance set at a *p*-value of < 0.05. The significance level was set after a Bonferroni correction for the between-group comparison of pain characteristics on the SF-MPQ. Calculations for effect size were then performed using Cohen’s d or *r* = z /√n for clinical characteristics with a significant between-group difference.

## 3 Results

### 3.1 Clinical and demographic data

**Figure 3:**
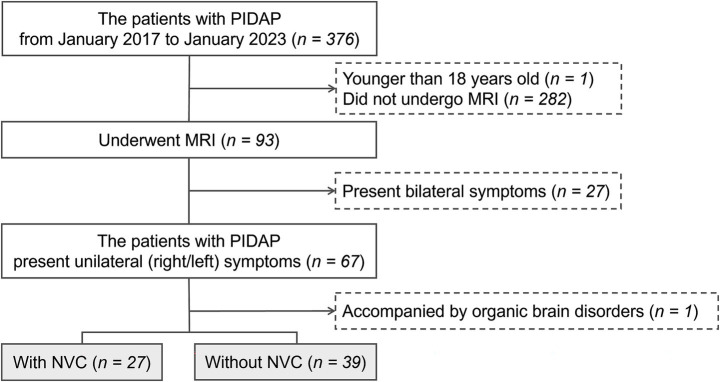
Flow of participants. In 376 new outpatients with PIDAP between January 2017 and January 2023, 93 patients underwent MRI. Following the exclusion criteria, the final eligible participants were 66 patients.

The final eligible participants were 66 patients (60 women, 6 men, mean age: 54.3 ± 14.0 years old, pain duration: 40.3 ± 47.9 months), accounting for 17.6% of the 376 new outpatients with PIDAP between January 2017 and January 2023 (Figure 3). NVC was detected in 27 patients: simple contact in 13, compression in 10, and displacement in 4; no NVC was observed in 39 patients. NVC on the ipsilateral side to the affected side was observed in 23 patients, contralateral NVC in 3, and bilateral NVC in 1. The participants were categorized into two groups: patients with PIDAP with NVC and without NVC, depending on the presence or absence of NVC.

**Figure 4:**
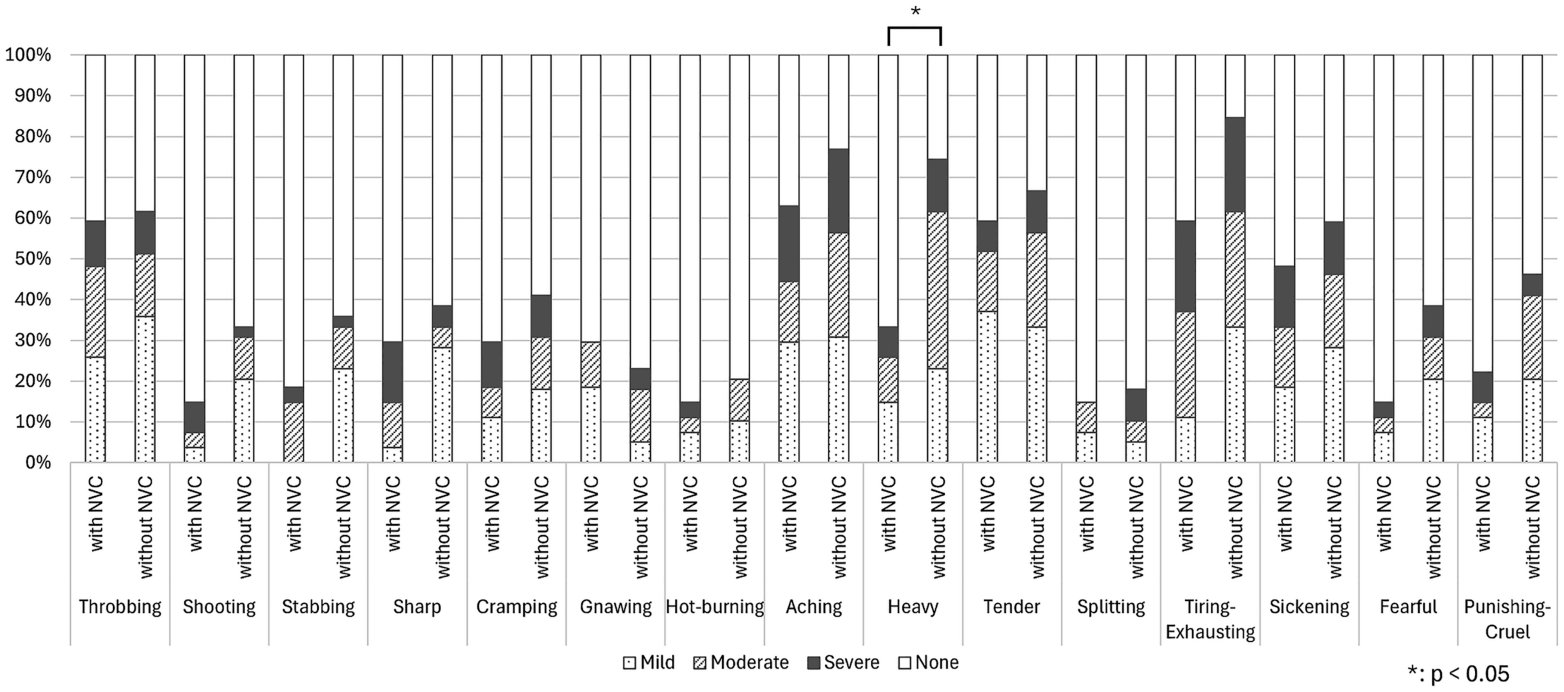
Differences in pain characteristics between the patients with PIDAP with and without NVC. Significantly more severe heavy pain was observed in the patients without NVC compared to that in the patients with NVC, using the short-form McGill pain questionnaire.

Patients without NVC showed significantly higher VAS scores (*p* = 0.024, effect size: 0.590, Table 1) and stronger heavy pain in SF-MPQ (*p* = 0.018, effect size: 0.402, Figure 4) compared to the patients with NVC. There were no significant between-group differences in other clinical data, including other pain characteristics (Figure 4), pain distribution, and the scores of SDS, PCS, and SSS-8 (Table 1), although affected pain characteristics: tiring-exhausting, sickening, fearful, punishing-cruel pain, in the short form of McGill pain questionnaires, seemed to be more severe in the patients without NVC than those with NVC.

**Table 1:** Differences in clinical and demographic data between the PIDAP patients with/without NVC.

Clinical characteristics	With NVC (*n* = 27)	Without NVC (*n* = 39)	*p*-value	Effect size	95% CI	
					Low	Upper
Sex (woman/man, woman ratio)	24/3 (88.9%)	36/3 (92.3%)	0.682	−0.058	0.124	3.583
Age	55.6 ± 14.7	53.5 ± 13.6	0.564	−0.147	−9.217	5.08
Duration of illness	29.7 ± 31.4	47.6 ± 55.8	0.102	0.377	−3.668	39.469
VAS	44.3 ± 27.5	58.6 ± 25.4	**0.024**	0.590	2.113	28.872
The presence of dental procedures as an onset trigger	17 (63.0%)	28 (71.8%)	0.592	−0.093	0.234	1.903
Root canal treatment	6	12				
Prosthodontic treatment	4	7				
Tooth extraction	4	5				
Restorative treatment	2	3				
Dental implant	1	1				
Pain distribution; laterality Laterality (right/left, right ratio)	14/13 (51.9%)	15/24 (38.5%)	0.459	0.096	0.550	4.010
Right upper molars	6	9				
Right upper incisors	1	3				
Right lower molars	6	5				
Right lower incisors	1	1				
Left upper molars	6	18				
Left upper incisors	2	1				
Left lower molars	8	9				
Left lower incisors	2	0				
The presence of psychiatric comorbidities	12 (44.4%)	17 (43.6%)	1.000	0.008	0.385	2.782
The presence of taking psychotropics or antiepileptics	9 (33.3%)	20 (51.3%)	0.208	−0.178	0.172	1.313
Antipsychotics	2	7				
Antidepressants	6	9				
Benzodiazepine anxiolytics or hypnotic	7	16				
Antiepileptics	1	5				
SDS	44.0 ± 8.2	45.6 ± 10.9	0.490	0.165	−3.059	6.321
PCS	28.8 ± 11.3	32.3 ± 11.0	0.220	0.312	−2.133	9.067
SSS-8	10.4 ± 6.7	8.7 ± 5.9	0.293	−0.273	−4.92	1.513

PIDAP, persistent idiopathic dentoalveolar pain; NVC, neurovascular compression; VAS, visual analog scale; SDS, Zung’s self-rating depression scale; PCS, pain catastrophizing scale; SSS-8, somatic symptom scale-8; CI, confidence interval. Bold numbers indicate *p* < 0.05.

### 3.2 Microstructural abnormalities at the trigeminal root entry zone

In patients with NVC, FA at the trigeminal REZ with compression was significantly lower than FA without compression (0.343 ± 0.050 vs. 0.390 ± 0.069; *p* = 0.006). Moreover, FA on the affected side of the trigeminal REZ was also significantly lower than FA on the unaffected side (0.333 ± 0.049 vs. 0.401 ± 0.059, *p* < 0.001). There was no significant difference in FA between the affected and unaffected sides in patients without NVC (0.361 ± 0.067 vs. 0.366 ± 0.048, *p* = 0.054). No significant difference in FA was detected between patients with and without NVC on the affected and unaffected sides (affected side: *p* = 0.060; unaffected side: *p* = 0.060).

### 3.3 Tract-based spatial statistics results

The TBSS analysis and subsequent analyses of white-matter tracts revealed no significant between-group differences. Despite the presence of NVC, the right-predominant FA pattern, showing higher FA in the right ROI than in the left ROI, was observed in the inferior longitudinal fasciculus, uncinate fasciculus, superior longitudinal fasciculus (temporal), and the left-predominant FA pattern was observed in the cingulum (cingulate gyrus) and inferior fronto-occipital fasciculus (Table 2).

**Table 2:** Significant right/left differences of FA in tracts and brain regions.

Region of interest	With NVC					Without NVC				
	FA	*p*-value	Effect size	95% CI		FA	*p*-value	Effect size	95% CI	
				Lower	Upper				Lower	Upper
Tract										
Anterior thalamic radiation R	0.456 ± 0.028	**0.018**	0.486	0.001	0.009	0.449 ± 0.025	< 0.001*	0.666	0.003	0.010
Anterior thalamic radiation L	0.461 ± 0.027					0.456 ± 0.027				
Cingulum (cingulate gyrus) R	0.489 ± 0.044	< 0.001*	1.830	0.052	0.081	0.494 ± 0.043	< 0.001*	2.045	0.051	0.071
Cingulum (cingulate gyrus) L	0.555 ± 0.037					0.555 ± 0.042				
Inferior fronto-occipital fasciculus R	0.501 ± 0.022	< 0.001*	1.030	0.007	0.015	0.492 ± 0.034	< 0.001*	0.810	0.006	0.015
Inferior fronto-occipital fasciculus L	0.512 ± 0.027					0.503 ± 0.034				
Inferior longitudinal fasciculus R	0.480 ± 0.018	< 0.001*	−1.035	−0.016	−0.007	0.467 ± 0.033	0.004*	−0.495	−0.011	−0.002
Inferior longitudinal fasciculus L	0.469 ± 0.020					0.460 ± 0.033				
Uncinate fasciculus R	0.507 ± 0.027	0.003*	−0.642	−0.022	−0.005	0.506 ± 0.038	0.005*	−0.447	−0.017	−0.003
Uncinate fasciculus L	0.493 ± 0.029					0.495 ± 0.036				
Superior longitudinal fasciculus (temporal part) R	0.596 ± 0.033	< 0.001*	−2.46	−0.099	−0.072	0.587 ± 0.031	< 0.001*	−1.872	−0.094	−0.067
Superior longitudinal fasciculus (temporal part) L	0.510 ± 0.030					0.507 ± 0.045				
Brain region										
Inferior cerebellar peduncle R	0.500 ± 0.030	0.005*	0.598	0.005	0.023	0.486 ± 0.043	< 0.001*	0.741	0.007	0.017
Inferior cerebellar peduncle L	0.514 ± 0.022					0.498 ± 0.040				
Superior cerebellar peduncle R	0.621 ± 0.024	< 0.001*	1.242	0.017	0.033	0.615 ± 0.027	< 0.001*	1.591	0.023	0.035
Superior cerebellar peduncle L	0.646 ± 0.025					0.644 ± 0.031				
Cerebral peduncle R	0.668 ± 0.028	0.915	0.021	−0.008	0.008	0.674 ± 0.026	**0.020**	−0.388	−0.009	−0.001
Cerebral peduncle L	0.668 ± 0.020					0.669 ± 0.026				
Anterior limb of the internal capsule R	0.590 ± 0.037	**0.024**	−0.461	−0.012	−0.001	0.582 ± 0.031	0.263	−0.182	−0.007	0.002
Anterior limb of the internal capsule L	0.584 ± 0.036					0.580 ± 0.034				
Posterior limb of internal capsule R	0.698 ± 0.020	0.200	0.253	−0.002	0.007	0.705 ± 0.026	**0.020**	0.390	0.001	0.007
Posterior limb of internal capsule L	0.701 ± 0.022					0.709 ± 0.027				
Retrolenticular part of internal capsule R	0.570 ± 0.019	< 0.001*	1.541	0.021	0.036	0.564 ± 0.030	< 0.001*	1.548	0.024	0.036
Retrolenticular part of internal capsule L	0.599 ± 0.019					0.594 ± 0.023				
Anterior corona radiata R	0.477 ± 0.030	0.012*	−0.521	−0.015	−0.002	0.467 ± 0.038	**0.031**	−0.358	−0.013	−0.001
Anterior corona radiata L	0.469 ± 0.034					0.460 ± 0.044				
Superior corona radiata R	0.505 ± 0.023	< 0.001*	0.833	0.006	0.018	0.508 ± 0.031	< 0.001*	0.695	0.006	0.018
Superior corona radiata L	0.513 ± 0.026					0.520 ± 0.039				
Posterior corona radiata R	0.489 ± 0.018	0.005*	−0.587	−0.016	−0.003	0.479 ± 0.035	< 0.001*	−0.575	−0.012	−0.003
Posterior corona radiata L	0.479 ± 0.018					0.471 ± 0.035				
Posterior thalamic radiation (including optic radiation) R	0.591 ± 0.025	**0.021**	−0.472	−0.013	−0.001	0.578 ± 0.044	0.011*	−0.43	−0.016	−0.002
Posterior thalamic radiation (including optic radiation) L	0.584 ± 0.029					0.569 ± 0.042				
External capsule R	0.415 ± 0.019	< 0.001*	1.794	0.025	0.039	0.407 ± 0.027	< 0.001*	2.452	0.031	0.041
External capsule L	0.447 ± 0.021					0.443 ± 0.031				
Cingulum (cingulate gyrus) R	0.506 ± 0.038	< 0.001*	1.586	0.036	0.059	0.511 ± 0.034	< 0.001*	1.886	0.033	0.046
Cingulum (cingulate gyrus) L	0.553 ± 0.036					0.550 ± 0.042				
Superior longitudinal fasciculus R	0.505 ± 0.017	0.124	0.306	−0.001	0.010	0.503 ± 0.031	< 0.001*	0.774	0.006	0.015
Superior longitudinal fasciculus L	0.509 ± 0.020					0.513 ± 0.028				
Superior fronto-occipital fasciculus (could be a part of anterior internal capsule) R	0.521 ± 0.034	< 0.001*	0.918	0.017	0.042	0.521 ± 0.038	< 0.001*	1.262	0.031	0.053
Superior fronto-occipital fasciculus (could be a part of anterior internal capsule) L	0.550 ± 0.037					0.563 ± 0.043				
Tapetum R	0.520 ± 0.052	< 0.001*	1.023	0.029	0.065	0.495 ± 0.075	< 0.001*	0.828	0.025	0.057
Tapetum L	0.567 ± 0.046					0.536 ± 0.073				

FA, fractional anisotropy, NVC; neurovascular compression, R; right side, L; left side, CI; confidence interval. Bold numbers indicate *p* < 0.05. *Significance with Bonferroni correction.

ROI-based analysis revealed no significant difference by Bonferroni correction; however, FA in some brain regions was lower in patients without NVC than in those with NVC (Table 3). On the point of right/left symmetry, many regions showed significant asymmetric patterns despite the presence of NVC as follows: the right-predominant asymmetry in the posterior corona radiata, and the left-predominant asymmetry in the inferior cerebellar peduncle, superior cerebellar peduncle, retrolenticular part of internal capsule, superior corona radiata, external capsule, cingulum (cingulate gyrus), superior fronto-occipital fasciculus, and tapetum (Table 2).

**Table 3:** Significant differences of FA in each brain region between PIDAP patients with/without NVC.

Tract/ Brain region	FA		*p*-value	Effect size	95% CI	
	With NVC	Without NVC			Lower	Upper
Affected/unaffected						
Sagittal stratum affected	0.528 ± 0.026	0.510 ± 0.037	**0.028**	−0.528	−0.033	−0.002
Fornix (cres) /stria terminalis Affected	0.526 ± 0.033	0.507 ± 0.040	**0.037**	−0.514	−0.037	−0.001
Fornix (cres) /stria terminalis Unaffected	0.527 ± 0.031	0.503 ± 0.045	**0.014**	−0.594	−0.043	−0.005
Right/left						
Sagittal stratum L	0.530 ± 0.030	0.512 ± 0.040	**0.039**	−0.500	−0.035	−0.001
Fornix (cres) /stria terminalis R	0.528 ± 0.031	0.507 ± 0.046	**0.028**	−0.525	−0.040	−0.002
Fornix (cres)/stria terminalis L	0.525 ± 0.033	0.503 ± 0.040	**0.018**	−0.589	−0.040	−0.004

PIDAP, persistent idiopathic dentoalveolar pain; FA; fractional anisotropy, NVC; neurovascular compression, R; right side, L; left side, CI; confidence interval. Bold numbers indicate *p* < 0.05.

### 3.4 The correlation between clinical features and FA

The partial correlation analysis, controlling for sex, age, pain duration and the presence of psychiatric comorbidity, on each tract and brain region revealed that the patients with NVC present negative correlations between the scores of SSS-8 and FA in the forceps major (*r* = −0.570, *p* = 0.005), left side of superior cerebellar peduncle (*r* = −0.545, *p* = 0.007), posterior thalamic radiation (*r* = −0.517, *p* = 0.012), superior fronto-occipital fasciculus (*r* = −0.566, *p* = 0.005), and tapetum (*r* = −0.594, *p* = 0.003), unaffected side of superior cerebellar peduncle (*r* = −0.525, *p* = 0.010) and superior longitudinal fasciculus (*r* = −0.528, *p* = 0.010), and affected side of superior fronto-occipital fasciculus (*r* = −0.622, *p* = 0.002), besides the SDS score and right side of superior longitudinal fasciculus (*r* = −0.562, *p* = 0.005) as shown in Table 4. On the other hand, in the patients without NVC, the scores of VAS indicating pain intensity positively related with FA in the affected side of uncinate fasciculus (*r* = 0.459, *p* = 0.006), left side of inferior fronto-occipital fasciculus (*r* = 0.437, *p* = 0.009), and unaffected side of the external capsule (*r* = 0.429, *p* = 0.010), and inferior fronto-occipital fasciculus (*r* = 0.427, *p* = 0.010); furthermore, the positive correlations were also observed in between the scores of SSS-8 and the not-affected side of anterior corona radiata (*r* = 0.470, *p* = 0.004) and uncinate fasciculus (*r* = 0.456, *p* = 0.006) and affected side of extra capsule (*r* = 0.493, *p* = 0.003) as well as in between the scores of PCS and right side of superior corona radiata (*r* = 0.431, *p* = 0.010), and left and affected side of uncinate fasciculus (*r* = 0.462; *p* = 0.005, *r* = 0.427; *p* = 0.011, respectively).

**Table 4:** Significant correlation between clinical characteristics and fractional anisotropy.

	Questionnaires	Region of interest	*r*	*p*-value
With NVC				
Tract	SDS	Superior longitudinal fasciculus R	−0.564	**0.005**
	SSS-8	Forceps major	−0.570	**0.005**
Brain region	SSS-8	Superior cerebellar peduncle L	−0.545	**0.007**
		Posterior thalamic radiation (including optic radiation) L	−0.517	**0.012**
		Superior fronto-occipital fasciculus (could be a part of anterior internal capsule) L	−0.566	**0.005**
		Tapetum L	−0.594	**0.003**
		Superior cerebellar peduncle unaffected	−0.525	**0.010**
		Superior longitudinal fasciculus unaffected	−0.528	**0.010**
		Superior fronto-occipital fasciculus (could be a part of anterior internal capsule) affected	−0.622	**0.002**
Without NVC				
Tract	VAS	Uncinate fasciculus affected	0.459	**0.006**
Brain region	PCS	Superior corona radiata R	0.431	**0.010**
		Uncinate fasciculus L	0.462	**0.005**
		Uncinate fasciculus affected	0.427	**0.011**
	SSS-8	Anterior corona radiata unaffected	0.470	**0.004**
		External capsule affected	0.493	**0.003**
		Uncinate fasciculus unaffected	0.456	**0.006**
	VAS	Inferior fronto-occipital fasciculus L	0.437	**0.009**
		External capsule unaffected	0.429	**0.010**
		Inferior fronto-occipital fasciculus unaffected	0.427	**0.010**

FA; fractional anisotropy, NVC; neurovascular compression, R; right side, L; left side, SDS; Zung’s self-rating depression scale, SSS-8; somatic symptom scale-8, VAS; visual analog scale, PCS; pain catastrophizing scale. Bold numbers indicate significance with Bonferroni correction.

## 4 Discussion

This study in the patients with PIDAP demonstrated four key findings: (1) the patients without NVC exhibited significantly higher pain intensity, particularly heavy pain in SF-MPQ, than those with NVC; (2) in the patients with NVC, FA in the trigeminal nerve at REZ was reduced, and negative correlations were observed between FA in the several brain regions and SSS-8 scores; (3) in the patients without NVC, there was no significant difference in FA at REZ, but FA in some brain regions were positively correlated with VAS, PCS, and SSS-8 scores; and (4) FA showed right/left asymmetric patterns in many tracts and brain regions, regardless of NVC presence. These findings represent the first investigation of both peripheral and central neural alterations in patients with PIDAP presenting unilateral symptoms, highlighting distinct differences in correlations between neural integrity, as measured by FA, and clinical features, depending on the presence or absence of NVC. This supports the hypothesis that PIDAP may comprise subgroups: one related to peripheral nerve impairment at REZ and the other potentially involving central microstructural alterations related to symptom severity, depending on the presence of NVC.

In patients with PIDAP presenting NVC, FA reduction at REZ, which contributes to peripheral nerve damage by NVC, may be involved. The current study of patients with NVC found significantly lower FA in the trigeminal nerve at REZ, particularly on the affected side, compared to those without NVC. This reduction in FA at REZ is indicative of demyelination, a phenomenon well-documented as the primary cause of typical trigeminal neuralgia (23). Thus, our findings suggest the existence of a PIDAP subgroup that involves peripheral demyelination by NVC at REZ. In addition to peripheral neural alterations, recent research has highlighted the involvement of central white matter alterations in pain integration, cognitive-affective processing, and motor function, even in primary trigeminal neuralgia, which was traditionally considered a purely peripheral disorder (4). In the present study, SSS-8 scores were negatively correlated with FA in regions such as the forceps major, superior fronto-occipital fasciculus, posterior thalamic radiation, and tapetum. FA decreased on the affected side of the superior longitudinal fasciculus. Forceps major and thalamic radiation, pathways for various sensory information, have been implicated as potential factors in chronic pain conditions (24). Previous studies in patients with trigeminal neuralgia have reported FA reductions in various brain regions, including the bilateral superior and anterior corona radiata, body, splenium, and genu of the corpus callosum, left cingulum, left superior fronto-occipital fasciculus, bilateral anterior and left posterior limbs of the internal capsule, left external capsule, left fornix cerebri, internal sagittal stratum, and left cerebral peduncle (25). Additionally, symmetric FA reductions have been observed in the corona radiata, internal capsule, optic radiation, and thalami (26). These involvements of central neural alterations alongside peripheral myelinations at REZ and the present study’s observations provide further support for the hypothesis that neural alterations at REZ may interact with reduced integrity in some brain regions associated with pain and somatic symptom burden in this patient population. Although additional investigations are needed to clarify this, the present study suggests that FA reduction in the trigeminal nerve at REZ and in some brain regions, which negatively correlates with somatic symptom burden, is involved in patients with PIDAP presenting with NVC.

On the other hand, the patients without NVC presented more severe pain compared to the patients with NVC, consistent with our previous report (7). Moreover, reduced neural integrity in some brain regions, including the region that links the right and left hemispheres, may be involved in patients without NVC. FA in the left sagittal stratum and bilateral fornix stria terminalis tended to decrease, as well as on the affected side of these brain regions, although no significance was detected. The sagittal stratum is a fronto-posterior fiber tract comprising several association and commissural fibers that play important roles in functions such as visual information processing, cognition, speech−/written-language, and spatial recognition (27). The fornix is generally known as a part that plays important roles in producing memories and emotions, connecting some regions of the limbic system. Furthermore, those regions play important roles in complicated connections between the right and left hemispheres. The study investigating FA differences in patients with chronic pain revealed widespread impairment of white-matter integrity, including the corpus callosum, internal capsule, external capsule, and thalamocortical pathway, suggesting that FA reduction in the interhemispheric and prefrontal-thalamic fibers is a common white-matter dysfunction in chronic pain (24). The reduction of FA in the sagittal stratum and fornix that relates to asymmetric dysfunction may result in more severe and complicated chronic pain sensations involving cognitive and emotional aspects of pain.

In patients without NVC, in addition to somatic symptom burden, pain intensity, and pain catastrophizing were positively correlated with FA in the uncinate fasciculus, anterior and superior corona radiata, external capsule, and inferior fronto-occipital fasciculus. The uncinate fasciculus anatomically connects the gyrus in the frontal lobe to the temporal lobe, and the corona radiata connects the cerebral cortex to the internal capsule. The inferior fronto-occipital fasciculus connects the prefrontal cortex with the occipital and temporal regions, taking important roles in cognitive and emotional functions. These regions, as well as the external capsules, have been considered in relation to pain sensations, including orofacial pain (4, 24–26). When considering neurological dysfunction, FA reductions indicating demyelination are generally focused on, and the clinical features of pain intensity and pain duration negatively affect FA in several brain regions in patients with chronic pain (24, 25). However, Kim et al. reported that upregulation of remyelination could be related, besides demyelination, to chronic pain mechanisms (28). Moreover, FA changes over time, depending on the extent of demyelination to remyelination (29). Longitudinal controlled studies are critical to investigate detailed mechanisms in FA alteration, including reflections of demyelination and remyelination, in pain-related brain regions and their interaction with clinical characteristics in patients without NVC.

Furthermore, the present study revealed right/left asymmetric patterns in many pain-related brain regions, independent of the presence of NVC. The previous report investigating the healthy human brain showed left-predominant FA in the posterior limb of the internal capsule and the cingulum, and right-predominant FA in the frontal lobe, including the uncinate fasciculus, posterior corona radiata, and posterior corpus callosum (30). Because the asymmetric patterns in the present study, especially in the posterior radiata, showed opposite asymmetry compared to previous reports, they may be related to the specific etiology of PIDAP. Posterior radiata are composed of nerve fibers that run from the cerebral cortex to the internal capsule and play an important role in the informative connection in the frontal–parietal–thalamus–brain stem system relating to orofacial pain sensations (4, 9, 24). Further studies comparing with healthy participants are crucial to elucidate whether the asymmetric FA patterns in pain-related regions could be a cause of PIDAP.

There are some limitations to this study. First, the principal limitation is that there are no healthy control subjects, but only comparisons among patients. This limitation prevents considering any pathophysiological mechanisms. Future controlled studies are essential to elucidate further mechanisms of PIDAP. In addition, the analysis, depending on the degree of NVC and pain characteristics, was not conducted due to the skewness of the sample size. The second is a cross-sectional study with a small sample size. This also limits the consideration of time-dependent mechanisms interacting with neural alterations. Moreover, as this is a retrospective investigation, the NVC assessment, which is based on clinical image diagnosis, was collected from medical charts without a sensitivity analysis. Further longitudinal studies with larger sample sizes that compare healthy participants are critical to elucidating the underlying central and peripheral nervous mechanisms in patients with PIDAP.

## 5 Conclusion

Further investigation is needed because no significant differences were observed in TBSS due to the small sample size and loss of controls. However, the results from ROI analysis yield additional clinical hypotheses. Patients with PIDAP presenting NVC may be associated with FA reduction in the trigeminal nerve at REZ and the central alteration, which relates to somatic symptom burden. On the other hand, patients without NVC, who report more severe pain sensations than the patients with NVC, may be associated with FA alterations in pain-related brain regions, which positively correlate with pain intensity and pain catastrophizing, besides somatic symptom burden. Further longitudinal controlled investigations are critical to clarify these differences and to determine whether asymmetric FA patterns in certain pain-related brain regions could relate to PIDAP regardless of the presence of NVC.

## Data Availability

The datasets presented in this article are not readily available because protection of patients’ privacy. Requests to access the datasets should be directed to Motoko Watanabe, totoompm@tmd.ac.jp.
